# Evaluation of the safety of C-spine clearance by paramedics: design and methodology

**DOI:** 10.1186/1471-227X-11-1

**Published:** 2011-02-01

**Authors:** Christian Vaillancourt, Manya Charette, Ann Kasaboski, Justin Maloney, George A Wells, Ian G Stiell

**Affiliations:** 1 Ottawa Hospital Research Institute, Clinical Epidemiology Program, The Ottawa Hospital - Civic Campus, 1053 Carling Avenue, Room F-658, Ottawa, ON, K1Y 4E9, Canada; 2 Department of Emergency Medicine, University of Ottawa, Ottawa, ON, Canada; 3 Regional Paramedic Program of Eastern Ontario, Ottawa, ON, Canada; 4 Department of Medicine, University of Ottawa, Ottawa, ON, Canada

## Abstract

**Background:**

Canadian Emergency Medical Services annually transport 1.3 million patients with potential neck injuries to local emergency departments. Less than 1% of those patients have a c-spine fracture and even less (0.5%) have a spinal cord injury. Most injuries occur before the arrival of paramedics, not during transport to the hospital, yet most patients are transported in ambulances immobilized. They stay fully immobilized until a bed is available, or until physician assessment and/or X-rays are complete. The prolonged immobilization is often unnecessary and adds to the burden of already overtaxed emergency medical services systems and crowded emergency departments.

**Methods/Design:**

The goal of this study is to evaluate the safety and potential impact of an active strategy that allows paramedics to assess very low-risk trauma patients using a validated clinical decision rule, the Canadian C-Spine Rule, in order to determine the need for immobilization during transport to the emergency department.

This cohort study will be conducted in Ottawa, Canada with one emergency medical service. Paramedics with this service participated in an earlier validation study of the Canadian C-Spine Rule. Three thousand consecutive, alert, stable adult trauma patients with a potential c-spine injury will be enrolled in the study and evaluated using the Canadian C-Spine Rule to determine the need for immobilization. The outcomes that will be assessed include measures of safety (numbers of missed fractures and serious adverse outcomes), measures of clinical impact (proportion of patients transported without immobilization, key time intervals) and performance of the Rule.

**Discussion:**

Approximately 40% of all very low-risk trauma patients could be transported safely, without c-spine immobilization, if paramedics were empowered to make clinical decisions using the Canadian C-Spine Rule. This safety study is an essential step before allowing all paramedics across Canada to selectively immobilize trauma victims before transport. Once safety and potential impact are established, we intend to implement a multi-centre study to study actual impact.

**Trial Registration:**

ClinicalTrials.gov NCT01188447

## Background

### Cervical spine injuries

**Figure 1:**
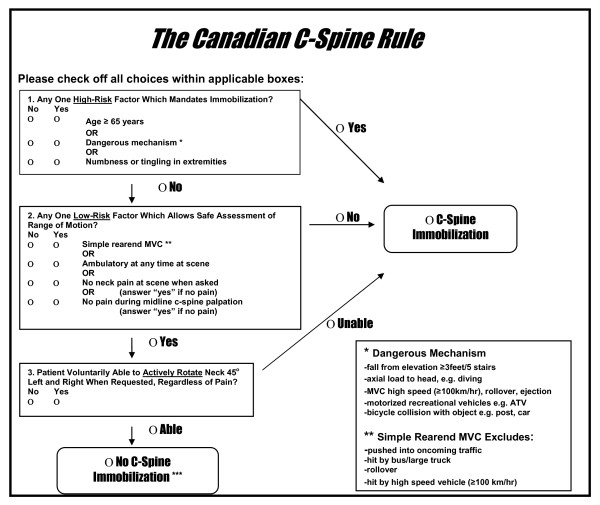
**The Canadian C-Spine Rule**. The Canadian C-Spine Rule for alert (Glasgow Coma Scale score 15) and stable trauma patients for whom cervical spine injury is a concern, including patients with either posterior neck pain with any blunt mechanism of injury or no neck pain but some visible injury above the clavicles. *MVC*, Motor vehicle crash.

Neck injuries are a common problem among blunt trauma victims with more than 8,000,000 cases being seen annually in U.S. and Canadian Emergency Departments (ED) [1]. While the majority of these cases represent soft tissue injuries, 30,000 patients suffer cervical spine fractures or dislocations and approximately 10,000 suffer spinal cord injury [2-4]. There are no readily available national Canadian data on ED visits such as those provided by the U.S. National Hospital Ambulatory Medical Care Survey [1]. The prevalence of potential neck injury can, however, be reasonably estimated for Canadian EDs. Extrapolation, on a population basis, from reliable U.S. figures [1] suggests that 1.3 million potential neck injury patients are seen annually in Canada. Only 0.9% of these patients are found to have cervical spine fractures or dislocations, even less (0.5%) have a spinal cord injury [5].

### Cervical spine radiography

Current use of cervical spine radiography for alert and stable trauma patients is very inefficient and highly variable. Most U.S. patients undergo radiography regardless of their clinical findings. Some maintain that all trauma patients should undergo such radiography [2,6-10]. This is mostly because of reports suggesting that clinical judgement alone is inadequate to predict injuries [6,11,12]. The American College of Surgeons recommends cervical spine radiography for all trauma patients with injury above the clavicle [7]. Indeed, a survey found that 97% of 125 U.S. trauma centres routinely order cervical spine radiography for all trauma patients [13]. In contrast, emergency physicians encounter a larger number of patients with very minor injuries, and some American emergency physicians are more selective about their use of cervical spine radiography.

Although selective use of cervical spine radiography is more common in Canada, we have shown that there is very large variation among hospitals and physicians in the use of radiography [14]. Universal cervical spine radiography has been considered inefficient by many authors who also note that the yield of this radiography for fracture or dislocation is very low [15-21], with the proportion of positive radiography being less than 3% in most trauma series [8,11,16-19,22-28]. Most authors suggest that radiography may not be required in alert patients with no pain or tenderness of the neck [4,11,15,16,18,19,21,22,25,29-36]. The huge number of normal cervical spine radiographs performed adds to health care costs [37] as well as to the burden of time and effort for emergency department staff, and Emergency Medical Services (EMS) who are expected to immobilize all patients before transport.

### Cervical spine immobilization

Because of the potential for spinal injury, paramedics go to great lengths to protect the cervical spine of trauma patients. Consequently, regardless of the presence of neck symptoms, most trauma victims transported to hospital in ambulances are protected by such measures as a backboard, collar, and head immobilization devices [4,22]. Not only is this often unnecessary, the potential for clinical adverse effects and discomfort from immobilization have been well documented. Chest straps used in immobilization have a marked pulmonary restrictive effect, even in healthy non-smokers [38]. Immobilization on a board leads to progressively worse pain in the head, neck, and back area, often resulting in the necessity to radiograph an otherwise normal spine in the ED [39-41].

Because trauma victims should be seen rapidly at the hospital, paramedics are given only 15-20 minutes to evaluate and treat them in the field before transport. Even for minor trauma victims, cervical spine immobilization takes at least five minutes to apply, or up to 30% of the allowed field time. Unlike minor trauma victims coming to the ED using their own means of transportation, who are most commonly sent immediately to the waiting room area, similar trauma victims immobilized and transported by paramedics can wait up to three hours until an ED stretcher becomes available, also holding up the EMS crew who then becomes unavailable for the next community emergency.

Once on an ED stretcher, it is not unusual for these patients to remain with full immobilization for several hours until c-spine radiographs or computed tomography can be performed and interpreted. As well, efforts to obtain satisfactory c-spine radiographs often require repeated attempts. This consumes valuable time for physicians, nurses, and radiology technicians and distracts them from other urgent responsibilities [15,42]. In addition, this delay compounds the burden of our crowded Canadian EDs in an era when they are under unprecedented pressures [42-44]. The median length of stay for a patient evaluated in the stretcher area is approximately eight to 12 hours, whereas similar minor trauma victims arriving without immobilization can be evaluated and discharged in less than four hours from the waiting room area.

### Clinical decision rules

Without the support of widely accepted guidelines, paramedics are likely to continue to immobilize all minor trauma victims. Clinical decision (or prediction) rules help to reduce the uncertainty of medical decision-making by standardizing the collection and interpretation of clinical data [45-48]. A decision rule is derived from original research and may be defined as a decision-making tool that incorporates three or more variables from the history, physical examination, or simple tests. These decision rules help clinicians with bedside diagnostic or therapeutic decisions. To fully develop a clinically effective rule is a lengthy process that involves separate studies to derive, prospectively validate, and finally implement the rule. The methodological standards for the derivation and validation of decision rules are well described [49-52].

Implementation to demonstrate the true effect on patient care is the ultimate test of a decision rule [53]. Unfortunately, many clinical decision rules are not prospectively assessed to determine their accuracy, reliability, clinical sensibility, or potential impact on practice. This evaluation is critical because many statistically derived rules or guidelines fail to perform well when tested in a new population [54-56]. The reason for this performance failure may be statistical, i.e., overfitting or instability of the original derived model [57], or may be due to differences in prevalence of disease or differences in how the decision rule is applied [58,59]. Most decision rules are never used after derivation because they are not adequately tested in validation or implementation studies [60-62].

### Previous guideline studies for use by physicians

In order to identify subgroups of trauma patients who need not undergo cervical spine radiography, many studies have been conducted in the past 15 years by emergency physicians [11,18,19,22,23,26,31,36,63,64], trauma surgeons [2,4,8,12,16,17,27,28,30,35,65,66], and radiologists [10,25]. Unfortunately, these studies suffer from great variability in design and none could be considered methodologically robust [67]. Of note are the U.S. based NEXUS Criteria which have received prominent attention after the publication of a huge validation study incorporating more than 34,000 patients [26,68,69]. Clinicians in Canada, however, have found several of the criteria to be poorly reproducible, namely "presence of intoxication" and "distracting painful injuries". Moreover, we recently attempted a retrospective validation of the NEXUS criteria based upon an existing database of 8,924 patients and found that the criteria missed ten of 148 clinically important injuries, yielding a sensitivity of only 93% [46]. We also found poor performance of the NEXUS criteria in our phase II prospective validation study [70]. We believe that the NEXUS criteria lack the accuracy and reliability to be useful for widespread clinical use.

### Previous guideline studies for use by paramedics

The necessity to immobilize all victims of blunt traumatic injuries during ambulance transport remains controversial. Despite the absence of difference in the neurological outcomes of 454 patients with blunt spinal injuries transported by a U.S. EMS system with full immobilization and Kuala Lumpur, Malaysia with no immobilization [71], most EMS systems continue to use back board, collar, and head immobilization during transport. We have been able to identify three original research papers that assessed the potential for paramedics to evaluate the c-spine in the field. Domeier conducted a large prospective cohort study evaluating selective immobilization by paramedics in 13,357 patients, 415 of which had cervical spine injuries [72]. Paramedics did not immobilize 33 of the 415 patients with spine injuries, none of which sustained a spinal cord lesion. Stroh retrospectively reviewed the health records of 861 patients transported to a trauma-receiving hospital using a selective immobilization strategy, and subsequently discharged with the diagnosis of cervical spine injury [73]. Five injuries were missed by their c-spine clearance protocol, one of which resulted in an adverse outcome. Muhr compared the immobilization rate in 293 patients before and 281 patients after the implementation of a selective spinal immobilization strategy, and found a 33% reduction in the rate of immobilization [74]. All three papers used the selective immobilization strategy described in the NEXUS studies. In Canada, the Canadian C-Spine Rule (CCR) is currently used in the city of Calgary and the province of Nova Scotia (without formal safety evaluation). Most other Canadian EMS are awaiting further safety evaluation studies before implementing such a program.

### Previous physician Canadian C-Spine Rule studies

The results of phase I, the derivation of the CCR, were published in *JAMA *in October 2001 [75]. This prospective cohort study involving 8, 924 stable, alert adult trauma patients was conducted in 10 large Canadian community and teaching hospitals (1996-1999). The ED physicians evaluated each patient for 20 standardized clinical findings and recorded these on a data sheet prior to radiography. Where feasible, a second physician conducted an independent interobserver assessment. Those variables found to be both reliable (kappa > 0.6) and strongly associated with the outcome measure (p < .05) were combined using recursive partitioning statistical techniques. The final model was formulated into a clinician-friendly algorithm, the Canadian C-Spine Rule (Figure 1). The rule stratifies patients into high-, medium-, and low-risk groups and requires evaluation of active range of motion for those in the low-risk group. This rule was cross-validated on the derivation sample and was found to identify all 151 cases of clinically important cervical spine injuries with a sensitivity of 100% (95% CI 98-100). The rule also performed with a specificity of 42.5% and would have required radiography for only 58.2% of patients, a 23.9% relative reduction from the current ordering rate of 76.5%.

The results of phase II, the validation of the CCR by physicians, were published in the *New England Journal of Medicine *in 2003 [70]. This prospective cohort study was conducted in nine large Canadian EDs (1999-2002) and enrolled 8,283 potential neck injury patients. More than 340 physicians explicitly and prospectively assessed patients for both the CCR and the NEXUS Criteria prior to diagnostic imaging and a second physician independently assessed some patients. The primary outcome, clinically important cervical spine injury, was defined as any fracture, dislocation, or ligamentous instability requiring internal fixation or treatment with a halo, brace, or rigid collar. The CCR was found to be highly sensitive for clinically important cervical spine injuries, identifying 161 of 162 cases. In the combined phases I and II, the rule would have identified 312 of 313 cervical spine injury cases, a sensitivity of 99.7% (95% CI 98-100). We also found the rule to be very reliable with a kappa value of 0.65. At the same time, our study found the NEXUS Criteria to have inadequate sensitivity, fair reliability, and very little potential to reduce use of radiography.

The potential impact on ED crowding was assessed by measuring the mean length of stay in the ED for patients without cervical spine injury and for whom reliable times were available. Patients who did not undergo radiography (N = 1,997) spent almost two hours less time in the ED (123.2 vs. 232.9 min; P < 0.001) than patients who did undergo radiography (N = 4,608).

We evaluated current physician practice, without the use of the CCR, by noting the number of cases where patients with cervical spine fractures were discharged from the ED without the fracture having been identified. This occurred 14 times during the study and nine of these cases were clinically important cervical spine injuries. All these patients returned due to ongoing pain or were recalled by the radiology department one or more days after the initial ED visit. Fortunately, no patient suffered an adverse outcome. In one of the nine clinically important cervical spine injury cases, no radiography was ordered during the initial visit. In another seven of the nine cases, physicians misread the radiographs as normal and the radiologists subsequently identified the error. In the ninth clinically important cervical spine injury case, the initial radiograph was actually normal.

Results from phase IIIa, which took place in 12 Canadian EDs from 2004 to 2006 (n = 11,824 patients) were recently published [76]. Phase IIIa was a matched-pair cluster design trial which compared outcomes during 12-month 'before' and 'after' periods at six 'intervention' and six 'control' EDs, stratified by teaching or community hospital status. All alert, stable adults presenting after acute, blunt head or neck trauma were enrolled. Sites were randomly allocated to either intervention or control groups. During the intervention-site after-period, active strategies were employed to implement the CCR into practice, including education, policy, and 'on-line' reminders. Outcomes included cervical spine imaging rates and missed injuries. From the before to after periods, the cervical spine imaging rate had a *relative reduction *of 12.8% at the six intervention sites from 61.7% to 53.3% (P = 0.01) but a *relative increase *of 12.5% at the six control sites from 52.8% to 58.9% (P = 0.03); this difference between groups was significant (P < 0.001). There were no missed c-spine injuries at the intervention sites. We concluded that, despite low baseline cervical spine imaging ordering rates, active implementation of the CCR by physicians led to a significant decrease in use of cervical spine imaging without missed injuries or patient morbidity. Widespread use of the CCR for clinical clearance of the c-spine could lead to reduced health care costs and more efficient patient flow in busy EDs.

### Validation of the CCR by paramedics

The validation of the CCR by paramedics took place between 2002 and 2006 in seven EMS systems distributed in three Canadian provinces [77]. The study population consisted of consecutive alert, stable, and cooperative adults transported by ambulance to the local lead trauma hospital after sustaining acute blunt trauma with potential injury to the neck. These are patients for whom standard basic trauma life support (BTLS) protocols require immobilization. Patient assessment was made by primary care or advanced care paramedics, who were trained by means of a 2-hour web-based training session followed by a practical demonstration to assess the CCR and the component clinical variables in a uniform manner. The paramedics recorded their findings along with their interpretation of the decision rule itself on a data collection form prior to arrival at hospital. They followed standard procedures for immobilization of patients and did not use the CCR as the basis for the decision to immobilize. The primary outcome, acute cervical spine injury, was defined as any fracture, dislocation, or ligamentous instability demonstrated on radiography. A clinically important cervical spine injury was defined as any injury requiring internal fixation or treatment with a halo, brace, or rigid collar. All enrolled patients who did not have radiography received telephone follow-up and were classified as having no acute cervical spine injury if they met all the previously validated explicit criteria at 14 days [78].

We enrolled 2,393 eligible patients in the study. 1,126 patients were not evaluated with radiography, and required telephone follow-up. We reached 788 (70.0%) of those patients, among which 682 were determined to not have sustained a cervical spine injury according to our validated proxy assessment tool. A total of 1,949 enrolled patients had complete outcome assessments. Twelve (0.6%) had a clinically important cervical-spine injury. In two cases, the investigators could perform an independent assessment of the rule based on the paramedic care report, but could not evaluate the paramedic assessment of the rule based on their study data collection sheet. The characteristics of the 444 patients without outcome assessments were similar to those with radiographic evaluation, but were less likely to be admitted to the hospital.

Paramedics conservatively misinterpreted the rule in 320 patients (16.4%), including 154 cases (7.9%) where "dangerous mechanism" was overcalled and 166 cases (8.5%) where paramedics did not evaluate neck rotation as required by the CCR. The CCR assessment for these patients was later categorized by the investigators as "indeterminate". Patient characteristics for these 320 patients where similar to those for which the rule was followed accurately, with the exception that none of the 320 patients had a cervical-spine injury. Paramedics did not attempt to evaluate neck rotation in any of the 12 patients with a clinically important injury. Excluding the indeterminate cases, the results yield a calculated sensitivity of 100% (95% CI 74-100%) and a specificity of 38% (95% CI 36-40). We performed secondary analyses involving all 1,949 patients to determine the potential effect of indeterminate cases when the rule was assessed by paramedics. When the rule was assumed to be positive for all indeterminate cases, the specificity was 32.4% (95% CI, 31 to 34), and when the rule was assumed to be negative for all indeterminate cases, the specificity was 46.6% (95% CI, 45 to 49). The sensitivity and negative predictive value remained the same since there were no cervical spine injuries among the indeterminate cases.

We assessed the reliability of paramedic interpretation of the rule among 155 paramedics by measuring the kappa coefficient for interobserver agreement for each element of the rule. The kappa value for the overall interpretation of the rule was 0.93 (95% CI, 0.87 to 0.99). In addition, agreement for the 8 individual components of the CCR was also very good, with kappa values ranging from 0.66 to 1.00.

The paramedics were asked to indicate on a five-point Likert scale how comfortable they would be in applying the CCR to this patient. The results were very supportive: Paramedics were "very uncomfortable" or "uncomfortable" applying the Canadian C-Spine Rule in 9.5% of cases; they were "comfortable" or "very comfortable" in 81.7% of cases.

We evaluated the potential impact of the rule on the number of necessary immobilizations. If paramedics were allowed to use the rule, 62.2% (95% CI, 60 to 64) of recruited patients would have required immobilization compared to the actual immobilization rate of 100%.

### Rationale for the study

We have previously derived (phase I) [75] and validated the CCR in physician (phase IIa) [70], ED triage nurse (phase IIb) [79] and in paramedic (phase IIc) [77] groups. We recently demonstrated successful implementation of the CCR by physicians in multiple hospitals (phase IIIa) [76], with a decrease in diagnostic imaging use by physicians and no adverse events. An implementation study using ED triage nurses is under way (phase IIIb). While we hope to demonstrate that ED triage nurses can safely remove patient's cervical immobilization devices, it would be significantly more valuable if we could empower the paramedics to selectively forego immobilization in the first place, and avoid great discomfort to patients. This is a practice already adopted by a number of U.S. and Canadian EMS services. We now hope to move the CCR project forward to the next level (phase IIIc) by carefully preparing paramedics to selectively immobilize the c-spine of very low-risk trauma patients who are alert and stable. Many decision rules in the past have not been widely adopted because of a failure to study implementation issues. We believe that this proposed safety evaluation study is an essential step towards the widespread implementation of the CCR by paramedics across Canada. If this evaluation study is successful, we can then plan wider dissemination of paramedic clearance in a future effectiveness trial. However, the current proposed study must demonstrate both safety and efficacy before dissemination can occur.

## Methods/Design

### Design

The proposed study will be a prospective cohort study comprised of a five-month training period followed by six-month run-in (could be shorter if no issues are identified) and 36-month evaluation periods in Ottawa, Canada. During the training period, paramedics will not actually clear the c-spine. During the 'run-in' and 'evaluation' periods, the paramedics will then be empowered by medical directive from the EMS medical directors and the Ministry of Health to "clear" the c-spine of patients according to the CCR. This will allow the paramedics to selectively transport low-risk trauma patients to the ED without full spinal immobilization. We will employ the run-in period immediately prior to the onset of the 'evaluation' period, to resolve logistical issues for the new practice of paramedics applying the CCR in the field. We will compare outcomes in the evaluation period of this study to those during the validation study at the same site (Ottawa) [77].

### Study population

#### Inclusion Criteria

We will enroll consecutive alert, stable adults evaluated by the paramedics with potential c-spine injury after sustaining acute blunt trauma. These are patients for whom standard EMS protocols require immobilization. Patient eligibility will be determined at the time of paramedic arrival at the scene based on the following criteria:

a) "Potential c-spine injury after sustaining acute blunt trauma" will include patients with either: i) neck pain with any mechanism of injury (subjective complaint by the patient of any pain in the posterior aspect of the neck),

ii) no neck pain but some visible injury above the clavicles, and/or

iii) neither neck pain nor visible injury, but significant mechanism of injury as determined by the paramedic at the scene.

b) "Alert" is defined as a Glasgow Coma Scale [80] score of 15 (converses, fully oriented, and follows commands).

c) "Stable" refers to normal vital signs as defined by the Revised Trauma Score [7] (systolic blood pressure 90 mm Hg or greater and respiratory rate between 10 and 24 breaths per minute).

d) "Acute" refers to injury within the past 4 hours.

#### Exclusion Criteria

a) Patients under the age of 16 years,

b) Patients with penetrating trauma from stabbing or gunshot wound,

c) Patients with acute paralysis (paraplegia, quadriplegia),

d) Patients with known vertebral disease (ankylosing spondylitis, rheumatoid arthritis, spinal stenosis, or previous cervical spine surgery), or

e) Patients referred from another hospital and transported between facilities.

#### Comparison Group from the Validation Study

We will quantify the potential impact of selective prehospital immobilization by way of comparison with a convenience sample of patients recruited in Ottawa during the validation of the CCR by paramedics between 2002 and 2006 [77]. These participants were recruited using the exact same criteria, and represent 862 of the 1949 recruited in the validation study [77].

#### Patient Safety

We are convinced that the use of the CCR is accurate and reliable and that the proposed study will respect patient safety at all times. Paramedics will know that they can 'override' the rule at any time when they have concerns about patient welfare. The CCR has proven to be very sensitive in identifying cervical spine injuries in previous validation studies with paramedics, nurses, and physicians and in the recent implementation study with physicians. With more than 32,000 patients evaluated, the CCR has never missed a single injury resulting in spinal cord injury. Nevertheless, we have included the following strategies to maximize the safety of this study:

1. We have re-designed the new proposal to include only a single centre to focus on safety and efficacy.

2. The single site proposed for this study also participated in the paramedic validation study.

3. Paramedics will only participate in this study if they:

• have completed a 2-hour training session and,

• pass a written test.

4. Paramedics will use the CCR under a medical directive signed by the Ministry of Health.

5. We will have an independent Data Safety Monitoring Committee review results on a regular basis; that committee will have the authority to recommend termination if patient safety is a concern.

#### Ethical Considerations

The study protocol received the approval of The Ottawa Hospital Research Ethics Board (protocol #2009142-01H) without the need for written patient consent. Paramedics will use the CCR under a medical directive, making training and participation in the study mandatory. Because of this, the Research Ethics Board also waived the need for paramedic consent. During a particular period in time, all eligible patients will be managed by the paramedics in the same manner in this observational cohort study. Patients will not be randomized. Patients will not be subjected to new therapy, invasive procedures, undue risk or discomfort, or investigations beyond that which would normally be required in the course of patient care. Patient confidentiality will be maintained throughout the study and patient names will be removed from all records. This is consistent with the approach approved by the Research Ethics Boards for our previous physician, ED triage nurse, and paramedic validation and implementation studies.

### Study interventions

#### Training

Initial training for all paramedics will entail one hour of self-review of a teaching CD, followed by a short quiz, followed by an in-person presentation of scenarios and question and answer. The teaching CD includes continuous audio and video presentation of slides on evidence and application, questions and answers, and case studies. All paramedics will have to successfully complete a written test in order to be certified in c-spine assessment.

#### Run-in period

This phase will allow the paramedics to fully clear the c-spine of low-risk patients. The purpose of the run-in period is to provide pilot experience with clearance and removal of immobilization in order to identify any logistical or unforeseen barriers. This run-in period is necessary since it will represent the first time that paramedics have actually not immobilized low-risk trauma victims in Ottawa, and we wish to proceed cautiously prior to enrolling cases for the study analysis.

#### Evaluation period

As in the run-in period, paramedics will be empowered by local medical directive to assess the c-spine of eligible patients according to the CCR and to selectively withhold immobilization where indicated. This will permit some patients to sit up on the ambulance stretcher and be taken to a low acuity or ambulatory area of the ED, rather than being left on a backboard in a high acuity or trauma area. Paramedics will record their findings on a simple Paramedic Data Form and in their paramedic ambulance care report.

#### Ongoing education

a) Study Champions will be identified among the EMS service. These "champions" will be paramedics who will serve as a local resource for the participating paramedics. The champion will be responsible for initial training, continuing education, and trouble-shooting issues brought up by the paramedics. They also helped recruitment significantly during the validation study.

b) Continuing education will be provided to all paramedics at least every six months and will be done by study champions in the format of small group sessions, such as during daily morning briefings, while the paramedics are on duty.

c) Newsletters will be developed and distributed to all study paramedics on a monthly basis. These will include tips on assessment and feedback on enrollment.

d) Local study champions will monitor staff turnover and ensure that new paramedics are trained for the study in a timely fashion.

### Outcome measures and data collection

#### Data collection

The following outcome data will be collected by dedicated study personnel who will review daily patient logs, ED patient records, diagnostic imaging reports, and in-patient records. All participating paramedics will complete a Paramedic Data Form at the time of each patient assessment as well as document patient care on their province wide Ambulance Call Reports. Additional study data will be recorded on a Case Record Form. All outcomes will be collected during the run-in and evaluation periods, but the run-in data will not be used in the final analysis (this run-in phase may be shortened if no issues are identified).

#### Measures of safety (primary study outcomes)

a) Number of missed cervical spine injuries, i.e. number of clinically important c-spine injuries (as defined previously) identified in patients who have had their c-spine cleared by paramedics. We will know the presence of fracture or cervical spine injury from review of diagnostic imaging reports in the patient record. We also propose to institute a strategy of surveillance to identify the uncommon occurrence of a fracture missed because no radiography was ordered. ED Patient Visit Logs at each receiving hospitals will be monitored for 30 days to identify return visits by patients who do not undergo radiography during their initial ED visit. In addition, we will review the Neurosurgery Patient Logs at our regional neurosurgical centre (The Ottawa Hospital, Civic Campus). This centre provides care for patients with spinal cord injuries for the whole Ottawa region. We recognize that there is a very small risk of not identifying a missed fracture but feel that this approach is pragmatic and feasible.

b) Number of serious adverse outcomes, i.e. development of neurological deficit after c-spine clearance by the paramedics. This very unlikely subset of missed cervical spine injury cases will be determined from review of the patient records. We will monitor for the extremely rare occurrence of motor weakness and disability that develops after paramedic assessment but do not expect this to occur.

#### Measures of clinical impact (primary study outcomes)

a) Proportion of low-risk patients transported without immobilization, i.e. proportion of eligible trauma patients who are not immobilized by paramedics. Daily EMS patient census logs will be reviewed to identify potential neck injury patients and then ED patient records (including ambulance call reports, nursing notes, and physician notes) will be assessed to determine eligibility. All eligible patients assessed by participating paramedics will be considered for the denominator of this measure. We will also report the number of eligible patients not assessed.

b) Lengths of time, i.e. time spent in the field before transport, time from ED arrival to transfer of patient care to ED staff; and total patient length of stay in the ED. These times will be compared, for those patients transported with and without spinal immobilization as part of the evaluation phase of this study, to those transported with immobilization (100% cases) during the validation study at the Ottawa site. We will only measure times for those patients who are assessed and enrolled by the paramedics.

#### Performance of the Canadian C-Spine Rule (secondary study outcomes)

The rule will be evaluated during the run-in and evaluation periods for all enrolled cases with completed Paramedic Data Forms.

a) Accuracy of the rule, i.e. sensitivity and specificity for identifying clinically important cervical spine injuries.

b) Paramedic accuracy in overall interpretation of the rule (immobilization required versus no immobilization required) will be determined by comparing the paramedics' response on the data collection form to the 'gold standard' interpretation of the rule made by the Investigators' Steering Committee. Attention will be focused on fractures missed or potentially missed by paramedic misinterpretation.

c) Paramedic agreement and comfort with and use of the rule. Paramedics, on the data collection form, will be asked to indicate their comfort in following the rule for each specific patient, using a five-point Likert scale. If the paramedic decides not to follow the rule, they will be asked to indicate reasons for their decision and if they recommend that additional follow-up and clarification from the study champion would be helpful. Ongoing education at regular intervals is a feature of the intervention and it will be important to address paramedics' concerns about using the decision rule.

### Data analysis

#### Measures of safety

We will list the number and describe the details of any cases deemed to be missed cervical spine injury or adverse outcome after clearance by the paramedics. The percent of missed cervical spine injuries will be estimated with point and 95% confidence intervals [CIs]. The estimates will be compared between validation and evaluation periods although we expect the missed injury rate to be 0% in both cases.

#### Measures of clinical impact

a) Proportion of low-risk patients transported without immobilization will be described as overall proportion with 95% confidence intervals, based upon a denominator of patients actually assessed by participating paramedics as judged by the completion of a Paramedic Data Form. This will be compared to the immobilization rate in the validation study, which we know to be close to 100% since paramedics were required to immobilize all patients by protocol.

b) Lengths of time will be presented as means plus standard deviations. We will compare time intervals for those patients assessed as part of the evaluation phase of this study, to those assessed during the validation study at the Ottawa site using the Student's t-test.

#### Performance of the Canadian C-Spine Rule

a) Accuracy of the rule: The classification performance of the rule for clinically important cervical spine injury will be assessed with 95% CIs for sensitivity, specificity, negative predictive value, and positive predictive value. The 'criterion interpretation' of the rule, i.e. positive or negative for cervical spine injury, will be made by the investigators based on the status of the patient for the component variables as documented by the paramedic.

b) Paramedic accuracy in overall interpretation of the rule: will be calculated as the simple agreement between the paramedics' responses on the data collection form to the investigators' 'criterion interpretation' of the rule.

c) Paramedic agreement and comfort with and use of the rule: these data for each individual patient will be tabulated in a simple descriptive format.

### Sample size

Sample size estimates for this study are governed by a number of considerations related to the various outcome measures (safety, clearance rate, accuracy) as well as feasibility. Our overall goal is to enroll patients in this evaluation study for 36 months, following the (up-to) six-month run-in period. Our future Phase IV implementation trial will have much larger patient numbers and more robust estimates of effect but we must demonstrate safety and efficacy first in this preliminary study. The results of this evaluation study will inform the design and sample size estimates for the future definitive Phase IV trial. Based upon the Paramedic Validation study, we expect that 380 paramedics will participate in the evaluation study and that 3,000 patients can be enrolled over 36 months. The consensus of the investigators is that this will give us an adequate number of cases in which to evaluate safety, clearance rates, and accuracy. We expect that 20 enrolled patients will have clinically important cervical spine injury. For clinical impact, we anticipate that as much as 40% of all patients assessed could be transported without full cervical spine immobilization.

## Discussion

We can expect paramedic use of the CCR to ultimately lead to improved efficiency for EMS systems, hospital EDs, and the Canadian health care system. Approximately 40% of all very low-risk trauma patients could be transported safely, without c-spine immobilization devices, decreasing the time spent in the field immobilizing patients before transport, and increasing paramedic field availability for the next patient from faster transfer of care to the ED personnel. While 1.3 million injury patients are transported each year by paramedics, the vast majority are low-risk and do not need cervical immobilization. This study is an essential step extending the responsibility of effective triage of trauma patients to paramedics across Canada. Most Canadian paramedics currently do not evaluate patients for potential c-spine injury, a task that is exclusively done by physicians. Our previous studies have determined the safety and effectiveness of the rule when used by physicians and nurses, but what remains unknown is safety and efficiency of patient care that would follow evaluation of the c-spine by paramedics. We believe that use of the CCR has the potential to increase the autonomy of the paramedic profession in managing the very common low-risk trauma patients. We expect the results of this efficacy study to be valuable and applicable to paramedics throughout all of Canada. We hope to plan a future implementation trial study that would focus on effectiveness in widespread Canadian locations. Our partners have not only expressed their support for this study, they have clearly indicated their intent to use the findings to change policies and guidelines within their organizations. These changes will eventually impact paramedic practice in all EMS services across Canada as well as in other countries.

## Competing interests

The authors declare that they have no competing interests.

## Authors' contributions

CV conceived the study and obtained funding. MC helped draft and edit the manuscript. AK obtained ethics approval. JM drafted and edited the medical directive and revised the methodology critically for important intellectual content. GW assisted with the methodology and revised it critically for important intellectual content. IS contributed significantly to the conception of the study and to the application for funding. All authors read and approved the final manuscript.

## Pre-publication history

The pre-publication history for this paper can be accessed here:

http://www.biomedcentral.com/1471-227X/11/1/prepub
